# Regulation of Emotions to Optimize Classical Music Performance: A Quasi-Experimental Study of a Cellist-Researcher

**DOI:** 10.3389/fpsyg.2021.627601

**Published:** 2021-04-06

**Authors:** Guadalupe López-Íñiguez, Gary E. McPherson

**Affiliations:** ^1^Sibelius Academy, University of the Arts Helsinki, Helsinki, Finland; ^2^Melbourne Conservatorium of Music, The University of Melbourne, Melbourne, VIC, Australia

**Keywords:** artistic research, emotions, expertise, intraindividual, linear regression, practice, professional musicians, self-regulation

## Abstract

The situational context within which an activity takes place, as well as the personality characteristics of individuals shape the types of strategies people choose in order to regulate their emotions, especially when confronted with challenging or undesirable situations. Taking self-regulation as the framework to study emotions in relation to learning and performing chamber music canon repertoire, this quasi-experimental and intra-individual study focused on the self-rated emotional states of a professional classical cellist during long-term sustained practice across 100-weeks. This helped to develop greater awareness of different emotions and how they vary over artistic events (9 profiled concerts and 1 commercially recorded album). Data analysis included traditional psychometric measurements to test the internal consistency of the time series data as well as the relationship between variables (artistic events). The study mapped the cellist’s flexible regulation of 17 different positive and negative emotions empirically linked to learning and achievement while practicing within the social context of performing music publicly at a high level. Findings arising from the study help with understandings of how to support musicians to maximize their artistic potential by reducing emotion dysregulation and strengthening the types of adaptive methods that enable them to manage their own emotions.

## Introduction

Both situational demands and individual differences impact the way humans regulate their emotions when engaged in demanding activities (Kobylińska and Kusev, 2019). The situational norms and socio-cultural context within which an activity takes place shape the types of strategies individuals will choose in order to regulate their emotions. Particularly interesting are the differences in how strategies are applied when attempting to regulate one’s emotions in a purposeful or proactive manner, or control them to cope with a challenging or undesirable situation (Kobylińska and Kusev, 2019), such as when a musician works to correct a lack of synchronization between the players, or instances where factors outside the control of the musician impact negatively on how the audience perceives the performance.

Individuals regulate their emotions differently. Whilst some responses are fixed or inflexible, and often maladaptive, healthy development involves “a series of challenges that require ever more sophisticated methods of adaptation including learning, self-regulation, and metacognition” (Hollenstein et al., 2013, p. 403). Effective emotion regulation results from an interaction between strategy-situation-personality patterns (Kobylińska and Kusev, 2019). An aim of recent research has been to understand these patterns more fully, in order to develop interventions that reduce emotion dysregulation and maximize adaptive methods of managing emotions (Kobylińska and Kusev, 2019).

## Theoretical Frameworks

### Flexible Emotion Regulation

One method of designing appropriate interventions has applied the concept of “flexible emotion regulation” to study how individuals are able to flexibly use different strategies, depending on current situational demands (Koole et al., 2015; Kobylińska and Kusev, 2019). This approach goes beyond the classification of different emotion regulation strategies as purely effective and adaptive or alternatively ineffective and maladaptive (Bonanno and Burton, 2014). Rather, it redefines the dimensions of emotion regulation according to a duality of flexible and adaptive, or alternatively lacking flexibility and not adaptive (Bonanno and Burton, 2014). Applying this perspective, researchers seek to find the best-fit situation-strategy patterns, in terms of what strategies are most effective and in what types of situations (see also Aldao, 2013; Aldao et al., 2015).

More recently, Kobylińska and Kusev (2019) have extended this conception to define “flexible emotion regulation as the ability to effectively regulate emotions by applying different emotion regulation strategies (chosen from a broad repertoire) in different situations depending on the features of a situation and one’s own personality characteristics” (p. 4). They suggest that strategies need to be tailored to cope with the demands of particular situations. Importantly, individuals vary in their sensitivity to the emotions they will experience in certain situations and the ease with which they will react by applying different approaches to cope effectively. Obviously, individuals who possess a broader repertoire of emotion regulation strategies will be better positioned to more flexibly implement adaptive strategies to cope with certain situations (Aldao and Nolen-Hoeksema, 2012a, b; Aldao et al., 2014). Likewise, differing strategies will be more or less effective, depending on the context (Sheppes et al., 2011).

The above framework can be applied to study the types of processes through which musicians modulate their emotions consciously and non-consciously as they respond to the changing demands of their musical environment (see also Goubet and Chrysikou, 2019). Social norms about how repertoire is to be performed, whether a particular interpretation will resonate with the audience (or a music critic) are just some of the ways that the context of a particular performance will influence what a musician feels before, during and after a performance. Musicians will also react differently to varying situations in the way they apply certain emotion regulation strategies to cope with a performance.

Previous studies have surveyed elite level performers to detail how they focus their thoughts and attention during high stakes performances. A good example is work by Buma et al. (2015) who suggest that elite level performers cope with the high level demands of performance by focusing their thoughts and attention on the physical aspects of the performance (e.g., breathing, pedaling), thoughts that give them confidence, and or a music-related focus. Their findings lead to questions of how to train young and emerging professional musicians to more purposefully apply these strategies in their development, especially for those musicians who experience performance anxiety or display maladaptive attributes.

To expand on work in both psychology and in music, this study documents the manner in which a professional cellist regulated her emotions as she learned and then performed and recorded demanding canon repertoire. Thus, the focus is on the types of strategy-situation-personality patterns of emotion regulation outlined by Kobylińska and Kusev (2019). To do this, the study details the thoughts and feelings experienced by the cellist across a 100-week period leading up to nine professional concerts and a commercially recorded album, and the manner in which these changed and evolved leading up to mastery of 150 min of repertoire.

### Self-Regulated Learning and Emotions

This study is part of a series of much broader series of studies that aim to document the type of behaviors, cognition and affect experienced by a professional cellist as she prepared works for multiple professional engagements. We chose as our framework the self-regulation triadic processes that are proactively as well as reactively adapted for the attainment of personal goals (Zimmerman, 2000). In keeping with the overall framework, we attempted to be sensitive to the influence of variations in context and personal experience (Zimmerman, 1989; Butler, 2002), given that the cellists’ personal capacity to self-regulate depends on her previous learning and development. Previous research by the authors have shown how external and internal aspects impact on the satisfaction of basic psychological needs and motivational resources across time (López-Íñiguez and McPherson, 2020); however, in this study we focus on the emotion regulation of the cellist.

**FIGURE 1:**
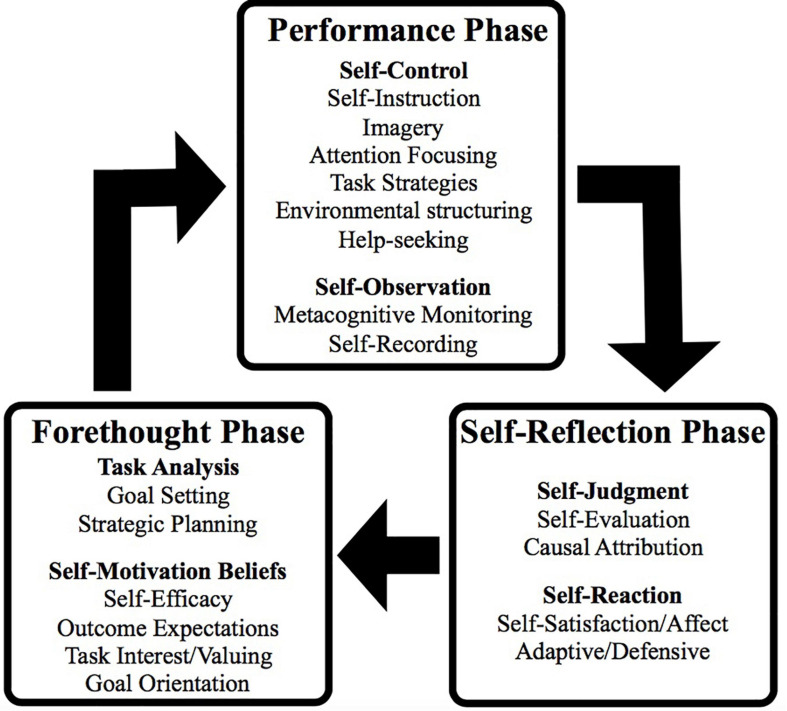
Phases and sub-processes of self-regulated learning (from Zimmerman and Campillo, 2003, p. 239).

Within the self-regulation literature, emotions are part of the forethought, performance and self-reflection phases (see Figure 1) of the self-regulated learning model:

• *Outcome expectations:* include the types of expected outcomes that precede one’s action (in contrast to outcomes that arise from our actions), and which are important for plans to change behavior. Outcome expectations involve being able to visualize an outcome and then determine whether you have sufficient capacity to perform this imagined outcome, and whether you believe that the outcome that you anticipate will occur because you performed (or failed to perform) a particular behavior.
• *Self-observation:* involves (a) *metacognitive monitoring* (i.e., tracking one’s own performance by observing and reflecting on what has been achieved and analyzing the results, relative to how you feel after completing the task); and (b) *self-recording* progress over time on the procedures that worked best whilst doing the task.
• *Self-reaction:* encompass how we give direction to our own actions and create self-incentives to persist and apply effort, by attending to (1) *self-satisfaction/affect* (our personal reactions to what we have just accomplished); (2) *adaptive* (inferences about prior performance that occur when one proactively sets about planning more effective strategies to be used); and (3) *defensive* (inferences that serve the function of protecting yourself from further dissatisfaction and failure that can result in negative emotions; see McPherson, in press).

Emotions (both positive and negative) are defined as short intense episodes that are different from moods of longer duration and stronger intensity (Rosenberg, 1998). In the emotion regulation literature (Dembo and Seli, 2012) research shows that positive emotions (e.g., hope, joy, pride) are related to self-regulation and high achievement, whereas negative emotions (e.g., anger, anxiety, boredom, depression, shame) appear to lead to more passive behavior and lower achievement (e.g., Pekrun et al., 2002, 2017; Pekrun, 2009; Goetz and Hall, 2013; Harley et al., 2019). One of the problems with research on this topic, however, is that self-reported emotional traits of individuals are challenging to reliably document via self-reports (e.g., Tapia, 2001), because of the subjectivity of emotional experiences (Robinson and Clore, 2002). In this study we report emotions based on self-perceptions and dispositions, which do not contradict the subjective nature of emotions, and for which objective selecting criteria were established.

### Emotion Regulation in Music

In music, a multitude of situations and individual personality patterns of reaction can produce negative emotions, especially when a concert or performance is impacted by influences outside the control of the performer, such as when an audience does not react in a manner expected or there is a lack of coordination among the musicians. Similarly, musicians can experience positive emotions when situations and individual personality patterns impact on their experience of flow within their performance, after receiving a standing ovation at the conclusion of a performance, or even after an individual practice session resulted in the mastery of a difficult section or feelings of greater confidence in being able to perform the passage more securely. Emotions are triggered in response to the type of situation and the way individuals respond by choosing strategies that are related to achieving specific goals. In these ways, emotions are important because they shape our behavior to facilitate or disrupt our goals and achievement.

The regulation of emotions in music has typically been studied from the perspective of listening to music in everyday situations to induce specific emotional states on young people (e.g., Thoma et al., 2012; Zhang et al., 2019; in mood regulation, see Saarikallio and Erkkilä, 2007; Stewart et al., 2019) or adults (Saarikallio, 2010), the neural mechanisms that underlie emotions regulation in music therapy treatments (e.g., Moore, 2013), the psychophysiological correlates of emotion in musicians’ arousal (Ogg et al., 2017), or in relation to the emotional states and emotional beliefs experienced by children musicians and teenagers (Kaleńska-Rodzaj, 2018, 2019) prior to public performances. Other research has focused on the mixed emotional experiences of musicians prior to performing on stage (Gabrielsson, 2001; Gabrielsson and Lindström Wik, 2003; Lamont, 2012), as well as how performers monitor and evaluate their emotional state immediately before a public performance, and consequently apply appropriate self-regulation strategies in order to change or maintain their emotions to desirable levels (Carver, 2004; Tamir, 2009).

Within the fields of music psychology and neuroscience, most of the studies concerned with emotions and musicians have focused on negative emotions such as anxiety during music performance (e.g., Matei and Ginsborg, 2017), as well as sadness and fear as experienced by musicians’ and non-musicians’ arousal (e.g., Park et al., 2014). (For an in-depth review of music and emotion studies, see Eerola and Vuoskoski, 2013).

In parallel with the above research, a number of studies have concentrated on academic subjects and how learners can be taught to change their emotions. Findings suggest that gifted and typically developing students differ exclusively in their expectancies for grades and success among a large variety of measures, including motivation (Kanfer and Goldstein, 1991; Ben-Eliyahu, 2019), or that people can be supported to achieve greater personal happiness, competence, and satisfaction through emotion regulation.

For the purpose of this study, we adopt methods from this literature to study the self-rated emotional states of a professional musician not only prior to public performing, but during long-term sustained practice, in order to develop greater awareness of these forms of emotion and how they vary over time. Our focus, therefore, was on the 17 different emotions that have empirically been linked to learning and achievement during the last five decades (Pekrun et al., 2002), and which, for the purpose of this study can be categorized across the dimensions of positive/negative or task or self-related/social:

• Positive Emotions:
  i. Task or self-related: joy, hope, enthusiasm, pride, hope, and relief
  ii. Social: gratitude, admiration, and surprise
• Negative Emotions:
  i. Task or self-related: hopelessness, sadness, disappointment, boredom, anxiety, and shame-guilt
  ii. Social: contempt, envy, and anger

Given the strong, longstanding research base in this area (Pekrun et al., 2002; Pekrun and Loderer, 2020), we chose to utilize these aspects of emotion to study high level skilled-based musical practice.

## Research Questions

Three questions guided the research:

• Immediately before practice sessions began (forethought phase), what negative and positive emotions were felt, and how did these feelings subsequently influence that practice session?
• Immediately after practice sessions had been completed (self-reflection phase), what negative and positive emotions were being felt at that time and remembered being felt throughout the practice session itself?
• To what extent did negative and positive emotions change from one practice session to the next, and overall, between each concert or album recording session?

## Materials and Methods

### Participant

This research was designed to document the processes experienced by an elite level musician using a research design in which a cellist was an active participant in the research who generated data as a result of her active participation in the social world being studied (e.g., Ellis and Bochner, 2000; Denzin, 2006). The advantages of employing this type of insider knowledge to reinforce research on expertise development has been emphasized in recent publications that defend the use of such approaches in order to “build a rich picture of phenomena exploring subtle social dynamics and interactions between individuals and employing organizations […] trying to understand what is happening, when, and why” (Yardley et al., 2020, p. 412).

Thus, this single-case study was focused on the first author, a professional cellist in her mid-thirties who is also a researcher in the educational psychology of music. As a female Spanish citizen residing in Finland, she is an active professional musician with a Master’s Degree in Classical Music Performance who specializes in solo recitals and chamber music concerts on period and modern cellos, and whose concerts and albums have been critically acclaimed internationally. In addition, she holds a Ph.D. in Psychology, with a specialization in constructivism in music learning and teaching.

### Design

A quasi-experimental approach (e.g., Cook and Campbell, 1979) examined within-person change and variability through an intervention that consisted of filling in surveys across individual practice sessions, rehearsals, concerts and during the recording of a commercial album. More specifically, the study examined whether and how such interventions (independent variable effects: artistic events and diary interventions) affected the repeated measures of the dependent variable (emotions), in trajectories of time (pre-, during-, post-rehearsals) and in different stages (concerts, album recording).

### Procedure

The study focused on intra-individual change and variability in emotion regulation whilst the cellist was learning canonic Classical-Romantic era works for cello and piano, and the way she optimized her performances over a period of 100 weeks (from 29/7/2016 to 19/10/2018) across 9 invited profiled concerts in Estonia, Finland, Germany, Russia and the United States, and a commercial album that took place between the last two concerts (Felix Mendelssohn: Complete Piano and Cello Workson Period Instruments; Alba Records ABCD 434). During this time, the cellist learnt and performed a total of 150 min of repertoire for the combination of cello and piano by Beethoven (Op. 5 Sonatas and all Variations) and Mendelssohn (the complete oeuvre), all of which are considered stylistically and technically demanding works.

### Forms of Data

For the purpose of the analyses reported in this article, forethought data included printed sheets including dichotomous items with two possible scores (yes/no) related to the types of emotion described above. Each of these were related to the question “What do you feel at the moment?” As this research was part of a larger data collection, the range of response variation was limited in order to not exhaust the cellist when collecting multiple data points every week for 3 years. The sheets included descriptions and examples of the seventeen types of emotions used in other research (Pekrun et al., 2002; Pekrun and Loderer, 2020) to cue the cellist’s self-reports before practicing: joy, gratitude, hopelessness, contempt, enthusiasm, admiration, pride, surprise, hope, sadness, disappointment, envy, relief, anger, boredom, anxiety, shame-guilt.

All inventories were adapted from the theoretical models by the researchers. Validity and reliability of the measurement techniques were provided by: (1) piloting the questions during five consecutive practicing days approximately 3 months before submitting the research proposal to carry out this project (which was subsequently funded); and (2) using the theoretical model to add descriptions and examples independently. For the self-reflection phase, data consisted of the same questions, including dichotomous items, as in the forethought phase so that the cellist could self-report about her emotions after practicing.

#### Processing of Data

Data collection involved recording information on average once a week. Precise days practiced and the repertoire selected for each concert are listed in Supplementary Material A. Data was transferred from printed sheets to machine readable forms that could be edited and then transferred to computer files for subsequent analyses. Total accumulated practice time for each of the days reported in this study ranged from 40 to 100 min per session (*M* = 65 min). Accumulated practice during other practice days where there were no diaries involved was not gathered. However, total practice estimates during the entire artistic project were gathered approximately five times more, given that the cellist practiced at least 6 days a week and logged her total practice estimates on approximately five other occasions during the entire artistic project. Locked filing cabinets were used to securely store source documents, and computer files were saved on the internal hard disk of a computer with password with an additional backup copy on an external disk with password. Both the computer and external back up were stored in a locked cabinet. Data was measured in terms of accuracy, timeliness and relevance, and transcribed immediately after each data collection point.

### Ethics

This study involves human participants and was reviewed and approved by the Ethics Committee at the University of the Arts Helsinki, Finland. Although the main report of data of this study concern the cellist-researcher, the pianists who played with the first author across this artistic research provided their written informed consent to participate in this study. In this article, pseudonyms have been used for all references to these musicians. In addition, written informed consent was obtained from the individuals for the publication of any potentially identifiable images or data derived from this research.

### Performance Approach and Instruments

As a cellist, the first author specializes in historically informed performance practice. Her performances adopted a musicological approach that includes visiting sites where the original and copyist manuscripts of the music and other miscellanea related to the composers were located (in Europe, the United Kingdom, and the United States; for more information, see López-Íñiguez, 2019). For this research project, the cellist played with three different professional pianists who performed on fortepianos and romantic pianos. A cello by Claude Pieray (Paris, 1725) with an instrumental set-up inspired by examples from the early 19th century, and classical-transitional bows by François Tourte (Paris, 1800) and by André Klaassen (Zutphen, 2015) were used across the project.

### Analysis

The focus of the analysis was on flexible/adaptive versus inflexible/maladaptive approaches to emotion regulation. This focus sought to map out the cellist’s (1) emotions in the forethought and performance phase, and (2) the flexibility of managing these emotions during and immediately after each practice or performance. Examples include situations where the cellist could (or could not) keep herself focused on her practicing/performing goals and the purpose of what she was trying to achieve regardless of experiencing negative emotions at the beginning of a practicing session, or artistic events that did not go as well as originally planned due to reasons that were or were not under the control of the cellist.

## Results

**FIGURE 2:**
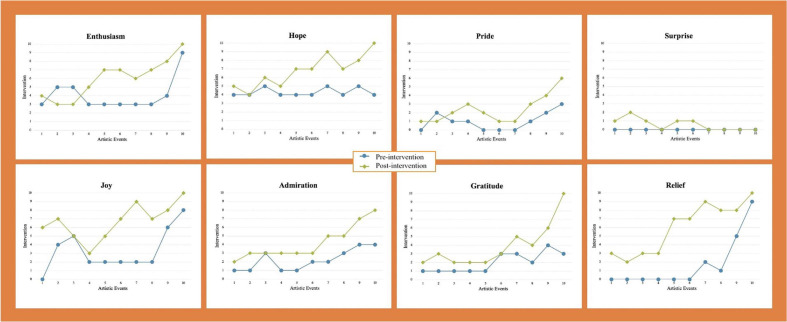
Visual direction for positive emotions. NB: Pre-intervention is represented by a blue line; Post-intervention is represented by a green line.

**FIGURE 3:**
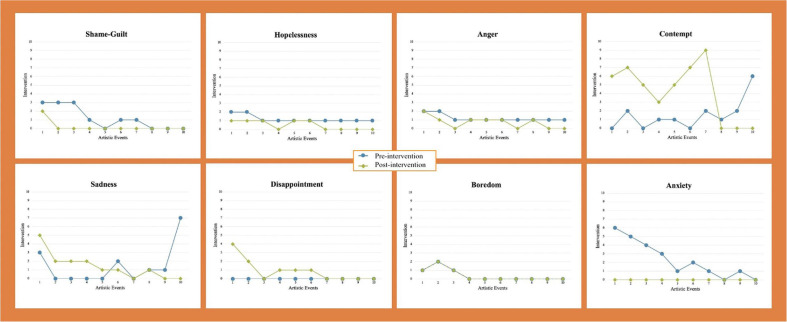
Visual direction for negative emotions. NB: Pre-intervention is represented by a blue line; Post-intervention is represented by a green line.

Figures 2, 3 below show the visual direction according to the scores (from 1 to 10 points) assigned to the questions based on Likert-type rating scales for positive and negative emotions regulation across concerts and the album (10 practicing sessions per artistic event). The scores can be observed in Supplementary Material B.

### Linear Regression

In addition, a linear regression was employed to test significant differences in the emotion items pre- and post-intervention and after all artistic events. For each emotion type, the pre-intervention and post-intervention scores were plotted against artistic event, to display the scores and help assess the pre-intervention versus post-intervention difference. A linear regression was performed for each, with emotion being the dependent variable and the independent variables being artistic events (understanding artistic event as a continuous variable on a scale from 1 to 10 study sessions) and intervention (pre-intervention versus post-intervention). Regression coefficients were estimated along with their associated standard errors, and *P*-values calculated to test the statistical significance of the coefficients (see Table 1).

**TABLE 1:** Effect of intervention and concert on positive and negative emotions.

	*p* Intervention	β Intervention	SE Intervention	*p* Concert	β Concert	SE Concert
Joy	0.001***	−3.4	0.832	0.008**	0.436	0.145
Enthusiasm	0.019*	−1.9	0.869	0.002**	0.452	0.730
Hope	0.000***	−2.6	0.483	0.002**	0.303	0.084
Relief	0.001***	−4.7	1.179	0.000***	1.045	0.205
Gratitude	0.002**	−1.7	0.452	0.000***	0.445	0.079
Admiration	0.000***	−2	0.422	0.000***	0.455	0.073
Surprise	0.007**	−0.600	0.194	0.023*	−0.085	0.034
Pride	0.015*	−1.4	0.516	0.009**	0.267	0.090
Anger	0.006**	0.600	0.190	0.001***	−0.133	0.033
Contempt	0.000***	−5.2	0.681	0.003**	0.406	0.119
Sadness	1	0.000	0.869	0.694	−0.061	0.151
Hopelessness	0.000***	0.700	0.154	0.001***	−0.112	0.027
Disappointment	0.021*	−0.900	0.354	0.018*	−0.161	0.062
Boredom	1	0.000	0.218	0.000***	−0.170	0.038
Anxiety	0.000***	2.3	0.517	0.002**	−0.324	0.090
Shame-Guilt	0.021*	0.700	0.275	0.000***	−0.288	0.048

*Indicates the level of significance.

### Narrative of the Turning Points

We used the critical/meaningful structural turning points of the graphics for the narration of results in connection to the external and situational context factors, as well as personal characteristics of the cellist (in depth analysis of these aspects in relation to motivation can be found in López-Íñiguez and McPherson, 2020), attending to:

1. the interaction between the cellist’s use of strategies, depending on the situation and her personal preferences and inclinations for working in a certain way (i.e., strategy-situation-personality patterns),
2. how her emotions were modulated or changed consciously and non-consciously to appropriately respond to environmental demands (i.e., how flexible emotion regulation was), and
3. the significant differences in the emotion items pre- and post-intervention and after all concerts (see the description of the linear regression procedure).

#### Positive Emotions

As depicted in Figure 2, there was generally a marked increase toward the end of the artistic project for the positive emotions of joy, enthusiasm, hope, relief, gratitude, admiration, and pride. This can be explained due to the cellist’s satisfaction with external and situational context factors such as the response of the audience and critics (social aspect), receiving external funding for the album and part of the concerts expenses when these were not covered by sponsors (task and social aspects), having two supportive pianists from the middle of the project onwards in comparison with the first pianist for this project with whom she did not feel artistically and personally aligned (task, self-related, and social aspects), and playing in acoustically superior and more profiled venues as the project developed (task aspect). These were also linked to internal aspects such as her progressive development of musical and technical mastery of the repertoire as the time spent practicing and richness of learning strategies increased (task and self-related aspects), as well as to personal characteristics such as being “mentally tough” (self-related aspect), and her self-determined focus to keep her feelings as positive as possible regardless of negative internal and external influences (task, self-related, and social aspects). Her self-determined focus was enhanced by providing herself with positive self-affirmation thoughts and choosing supportive activities such as yoga, meditation, osteopathy, and maintaining social contacts through meetings with friends (task, self-related, and social aspects). In addition, a strong disposition for becoming a better learner regarding emotion regulation and its effect on overall performance during the entire project was fostered by continuously striving to understand how emotions about herself – before, during and after the practice sessions—were interconnected to her own actions, thoughts and feeling, in addition to the actions of other people.

The cellist felt less energized with a decrease in positive emotions during practice sessions and after concerts leading up to and including Concert 3, when personal and professional issues started to emerge with her first pianist, and she became increasingly aware of the need to find a new partner for the rest of the project. This can be contrasted with the extremely positive emotions experienced in Concert 7 when both practice preparation and the performance was of a higher quality than in previous concerts. Her developing friendship with a new pianist exerted a positive impact on how she felt post-interventions. Overall, her feelings of joy and enthusiasm increased during this stage of the project because she felt much more positive about the overall artistic journey and the possibility of successfully completing a project of this type. It was during this period that she also received significant additional external funding for her album expenses. In addition, the intensity of the emotion surprise decreased toward the end of the project because the cellist felt she had fulfilled her artistic vision for the repertoire being performed. In fact, at this time she felt what she refers to as a sense of artistic “emptiness” and, even though she felt content with what she had accomplished, she was becoming more interested in future and rather different artistic projects. According to all artistic events and the 10 rehearsal sessions within each event, a linear regression found significant differences in the positive emotions pre- and post-intervention and after all events (including the album recording) (see Table 1).

As a last aspect in relation to the positive emotions, two important turning points in the project were the last two concerts (events 8 and 10) and the recording of half of this repertoire for a commercial album in between these two concerts (event 9). In her opinion, the final two concerts were the best artistically for the whole project and this was confirmed by an expert musician who followed all these events, the musicians themselves, and the non-systematic way of collecting feedback from the audience and critics. Recording the music, which for a musician can be a physical and emotional energy-consuming activity, led to a feeling of finality for the project.

#### Negative Emotions

According to the negative emotions, Figure 3 showed that anger, contempt, hopelessness, disappointment, sadness, boredom, anxiety, and shame-guilt decreased across the project^1^. As confirmed in a linear regression on these emotions, significant differences were found between pre- and post-interventions and after all concerts for anger, contempt, hopelessness, disappointment, anxiety, and shame-guilt. However, non-significant differences were found for sadness overall, and for boredom what comes to the practicing sessions. This parallels the results in the positive emotions of surprise, joy and gratitude because the cellist was positively involved in her “dream” artistic project (task and self-related aspects), with the result that there were few opportunities or instances where she felt bored or sad.

The results related to negative emotions can also be explained in relation to the abovementioned cellist’s personal characteristics, the situational contexts and interpersonal aspects for the project. For instance, although the cellist generally started the sessions feeling more negative than positive (see Supplementary Material B for the scores), all negative emotions markedly decreased after the practicing sessions and artistic events, except for sadness, which despite not being significant in the linear regression, showed a marked increasing tendency for the last concert (event 10), as this was the end of the project for which the cellist had dedicated so much energy, ideas, resources, and time, and where she felt “mentally, physically and artistically exhausted” (task and self-related aspects). The experience of feeling more negative emotions at the beginning of the project and before practicing sessions can be explained by the cellist not feeling entirely confident with the new repertoire and the first rehearsals with her new accompanist, and a feeling that she could have been better prepared for the first 3 concerts in particular (task, self-related, and social aspects).

Finally, in the earlier stages of the project, the cellist experienced intense feelings with regard to the project, due to the amount of time practicing and reading different sources, setting up her instrument, solving certain muscle issues with specialists, finding funding and performance opportunities in addition to other work demands. These task-related, self-related and social aspects impacted her negative emotions in particular. From the middle of the project onwards, when these aspects had been solved or mastered, she experienced a wider range of both positive and negative emotions.

#### Overall Emotion Regulation

Up until the middle of the project the cellist felt that she was reacting more strongly to external influences, and because she was not always able to control these, she felt less in control of events as they unfolded. However, these feelings drove her to feel that she needed to work even more intensively to maintain a positive attitude. From the middle of the project, she experienced a shift toward internal aspects such as her own artistic and personal development. This shift resulted in her feeling more in control of the project, and more able than in earlier stages to flexibly regulate her emotions to experiencing positive emotions. She attributed her increased ability to regulate her emotions as being partly due to the constant use of self-reports and self-regulating learning strategies.

## Discussion

In the field of music, there has been an emphasis in studies dealing with cognitive processes and behaviors and within this, in particular, the importance of self-regulation of motivation among musician experts (e.g., Woody and McPherson, 2010; Evans and McPherson, 2017; López-Íñiguez and McPherson, 2020). Motivation has been typically seen as a psychological feature that seemingly shares cause-and-effect relationship with the emotions of people–for instance, emotions can have detrimental implications on overall achievement (see the cognitive-motivational model on the effects of emotions by Pekrun, 1992; also Tamir, 2016) by undermining intrinsic motivation (Pekrun and Perry, 2014).

The results of this research, particularly the turning points that aligned with certain types of emotions, are similar to other work by the authors showing how external and internal aspects impact on a cellist’s satisfaction of basic psychological needs and motivational resources across time (López-Íñiguez and McPherson, 2020). In this particular study, however, we followed the most recent international discourse of emotions as crucial influences on learning and achievement (e.g., Pekrun and Linnenbrink-Garcia, 2014; Keefer et al., 2018), for which it has become increasingly relevant to study the (self-) regulation of emotion in overall academic settings (Ben-Eliyahu, 2019), including music. Specifically, we analyzed whether the intensity of positive and negative emotions decreased or increased during and across a period of 100 weeks by a single professional cellist, as she prepared for performances and a commercially recorded album.

The study shows evidence of flexible emotion regulation over time by a professional classical musician, both according to the situational context of the study, as well as to the different tasks and self-related/social dimensions involved in it, as described by Kobylińska and Kusev (2019). The research is part of a much larger series of investigations that are documenting the processes of a single professional musician (e.g., López-Íñiguez and McPherson, 2020). Our results show that the cellist focused on her goals and was unwilling to give up. She was also able to maintain a belief in her abilities and used sustained self-regulating strategies over a long period of practice sessions and performances (100 weeks) to make changes in the desired direction of her emotions. These were adapted according to the various complexities she faced in each practice and performance situation. These results connect also to studies showing that experts are more flexible in their use of strategies, depending on the performance situation (Bilalić et al., 2008; Kobylińska and Kusev, 2019).

The cellist generally used positive thoughts while practicing by constantly reminding herself that she could solve technical problems arising during practice sessions or after performances, as well as deal with personal and professional dissatisfactions during the entire process. These results are similar to those found in studies on successful student learning showing that feelings of shame in academic settings are reduced as the learner becomes increasingly goal-oriented and feeling in control of their learning (Turner et al., 2002). In particular, the main aspects influencing positive and negative emotions were represented by both external/internal factors (e.g., developing friendships with the pianists, receiving positive/negative feedback and criticism, feeling satisfied/dissatisfied with her own musical/technical learning and performance progress, personal characteristics such as being tough, determined and persistent, receiving funding and financial support; playing in more or less acoustically rewarding places) and self-regulatory strategies used (e.g., continuously filling up the self-report measures despite being sometimes tired, using positive self-thoughts in any positive/negative situation or when confronted with challenges, using meditation, yoga and osteopathy, and progressive control of the overall project through increasingly better planning and assessment of learning processes).

In addition, persistence and disposition toward learning more about her emotions, and flexibly regulating them toward more positive emotions through the use of self-reports, shaped the way the cellist pursued and refined her own identity as a learner (Coll and Falsafi, 2010). This is similar to the type of learning mindset characteristics of learner-centered education in music (López-Íñiguez et al., 2020), and other evidence that associates performing musicians’ successful careers and their overall enjoyment (López-Íñiguez and Bennett, 2020), and motivation (López-Íñiguez and McPherson, 2020). These factors allow a musician to thrive emotionally. In fact, it has been argued that when learners take on additional responsibility for any aspect related to their own learning – in this case emotions – their identity as independent lifelong learners is further strengthened (Kolb and Kolb, 2012).

## Conclusion

In light of the results of this study, we suggest that emotions should be considered as important objects of learning, beyond the typically studied cognitive, behavioral and motivational aspects present in the mainstream expert literature (in line with Ben-Eliyahu and Linnenbrink-Garcia, 2015; Ben-Eliyahu, 2017). We agree with Ben-Eliyahu (2019) “that teaching learners to regulate emotions to facilitate potential benefits during intellectual work can and should be an explicit target during learning, alongside intellectual academic skills” (p. 3).

In line with this, our research suggests that experts and aspiring musicians are better placed if they possess a toolkit of emotion regulation strategies to cope with demanding situations because self-regulating one’s own learning may give rise to positive feelings, whereas external control may induce boredom, anxiety, or anger (Titz, 2001; Pekrun et al., 2002). In this way, musicians could go beyond techniques such as meditation to positively alter their mood and emotions (e.g., Bourne, 1995) toward a self-regulating learning strategy such as the employed in this study that has proven extremely successful in music studies. Furthermore, this can result in a broader repertoire of strategies to regulate one own’s emotions and thus cope with demanding situations (Aldao and Nolen-Hoeksema, 2012a, b; Aldao et al., 2014).

Another important dimension concerns how certain emotions experienced by performers when practicing or performing can result in different levels of motivation and alternatively how being motivated can cue or elicit certain types of emotions. For instance, when the cellist read positive feedback from the critics or the audience, she experienced gratitude and pride, which consequently motivated her to value her efforts and continue practicing (identified regulation). On the other hand, despite feeling hopeless due to muscle issues or disappointed because of the reported complex social dynamics, her passion for the chosen repertoire and enjoyment on stage kept her going (intrinsic motivation). Such two-way interactions between motivation and emotion have rarely been addressed in studies involving the measurement of emotions or moods experienced in musicians over time but would contribute to current understandings of musical learning and performance.

The research documented here goes beyond the limitations found in retrospective self-report measures that are frequently used in self-regulated learning studies (see McPherson et al., 2019). We acknowledge, however, that our approach does have certain limitations that are typical of quasi-experimental designs (e.g., Shadish et al., 2002). One disadvantage is that longitudinal studies are challenging logistically when studying multiple (and randomized) elite-level musicians. Part of the challenge is that self-regulation tasks are cognitively demanding and participating musicians can be unwilling to devote sufficient time and energy to mapping their own practice and professional engagement habits. Despite being restricted to a single cellist, our findings do concur with studies involving larger samples of musicians (Osborne et al., 2020) indicating that encouraging learners to engage more often with self-regulation tasks can help to reduce their emotion dysregulation.

Further studies might include measuring levels of cortisol secretion and performance success to refute or support self-report evidence, using, for example, techniques that have been developed to measure tennis players’ reactions to pressure (Laborde et al., 2014). Other approaches might include collecting data from open-ended questions including extensive narrative details of how emotions change over practice or studying how emotions are connected to other areas of music learning and performing. In this regard, our study is part of ongoing attempts to study the different aspects that affect a professional musician’s practice. Our work has investigated motivational aspects (López-Íñiguez and McPherson, 2020), but also plans to target the role of quantity and quality of practice (following Ericsson and Pool, 2016) in connection with the use of learning model of conditions, processes and results during practice (following Pozo et al., 2020). Such work will attempt to further knowledge of the psychological aspects that underlie expert performance.

## Data Availability Statement

Data is not available in any open data repository. A selection of the pseudonymized datasets generated for this study are available on request to the corresponding author.

## Ethics Statement

The studies involving human participants were reviewed and approved by the Ethics Committee at the University of the Arts Helsinki, Finland. The patients/participants provided their written informed consent to participate in this study. Written informed consent was obtained from the individual(s) for the publication of any potentially identifiable images or data included in this article.

## Author Contributions

GL-Í conceived the artistic project, undertook the initial designing of the study, undertook the experiment and artistic project, collected, transcribed, and coded the data, performed all analyses, and also wrote the first draft of the manuscript with support from GM in all sections. GM developed and adapted the theoretical framework used to underpin the study, formulated the conceptual ideas adapted in the manuscript, supervised the findings and statistical analysis, contributed to the interpretation and presentation of results, and provided language support, literature advice, and writing advice to GL-Í. Both authors provided critical feedback at all phases, discussed the results, and assisted each other to complete the final version of the manuscript.

## Conflict of Interest

The authors declare that the research was conducted in the absence of any commercial or financial relationships that could be construed as a potential conflict of interest.

## Footnotes

1 The emotion *envy* has not been included in the analysis as the cellist never selected it before or after the study sessions.
