# Supplementary material for: Regulation of Emotions to Optimize Classical Music Performance: A Quasi-Experimental Study of a Cellist-Researcher
Source: Front Psychol. 2021 Apr 6;12:627601. doi: 10.3389/fpsyg.2021.627601 (PMC8056010; doi:10.3389/fpsyg.2021.627601)
Supplement: Supplementary file 1 [file Table_1.pdf]

Supplementary Material A

List of concerts, practicing days, programs and keyboards employed

Year	Concert Day and Place	Pre-Concert Practicing Days	Program	Keyboard employed
2016	4 October / *Sellosali* Concert Series, Espoo (Finland)	29.7; 5.8; 12.8; 19.8; 26.8; 2.9; 9.9; 16.9; 26.9; 30.9	-Beethoven: Sonata Op. 5, N.1; Variations WoO45; Variations Op. 66<br>-Mendelssohn: Variations Concertantes; Sonata D Major	-Replica of an Anton Walter fortepiano from 1795, built by Michael Walker, Germany<br>-Original Clementi fortepiano (London, 1828)
	1 November / *Kloostri Ait*, Tallinn (Estonia) & broadcasted by Klassikaraadio in Estonia	6.10; 10.10; 13.10; 15.10; 17.10; 20.10; 22.10; 24.10; 27.10; 29.10	-Beethoven: Sonata Op. 5 N. 1; Variations WoO45; Variations Op. 66	-Replica of an Anton Walter fortepiano
2017	24 May / *Nordic Historical Keyboard Festival*, Kuopio (Finland)	17.3; 24.3; 31.3; 7.4; 14.4; 21.4; 28.4; 5.5; 12.5; 19.5	-Beethoven: Sonata Op. 5, N.2; Variations WoO45; Variations WoO46; Variations Op. 66	-Replica of a five-octave Stein fortepiano made by Pentti Pelto
	12 September / *Aino Ackté Festival*, Helsinki (Finland)	7.7; 14.7; 21.7; 28.7; 4.8; 11.8; 18.8; 25.8; 1.9; 8.9	-Beethoven: Sonata Op. 5 N.2; Variations WoO46<br>-Mendelssohn: Variations Concertantes; Sonata D Major	-Replica of a Graf's fortepiano from 1820, built by Rodney Regier, United States
	28 November / *Uusi Paviljonki* Concert Series, Kauniainen (Finland)	22.9; 29.9; 6.10; 13.10; 20.10; 27.10; 3.11; 10.11; 17.11; 24.11	-Mendelssohn complete piano and cello works	-Original Érard piano (Paris, 1862), serial number 32602 (AAA-a''''), una-corda and damper pedals
2018	18 April / *Beethoven Research Center*, San José, California (USA)	9.2; 16.2; 23.2; 2.3; 9.3; 16.3; 23.3; 30.3; 6.4; 13.4	-Beethoven: Sonata Op.5, N.1; Sonata Op. 5, N.2; Variations WoO45	-Original Broadwood fortepiano from 1823 (CC to f'''')
	15 July / *Piano Salon Christophori*, Berlin (Germany)	11.5; 18.5; 25.5; 1.6; 8.6; 15.6; 22.6; 29.6; 6.7; 13.7	-Mendelssohn complete piano and cello works	-Original Érard piano from 1854
	8 August / *BRQ Vantaa Festival*, Vantaa (Finland)	17.7; 19.7; 21.7; 24.7; 26.7; 28.7; 31.7; 2.8; 4.8; 6.8	-Mendelssohn complete piano and cello works	-Original Érard piano (Paris, 1862), serial number 32602 (AAA-a''''), una-corda and damper pedals
	16-17 August and 1-2 September 2018 / CD Recording, Kauniainen (Finland)	18.6; 25.6; 2.7; 9.7; 16.7; 23.7; 30.7; 13.8; 20.8; 27.8	-Mendelssohn complete piano and cello works	-Original Érard piano (Paris, 1862), serial number 32602 (AAA-a''''), una-corda and damper pedals
	25 October / *Philharmonic Society's Small Hall*, Moscow (Russia)	17.8; 24.8; 31.8; 7.9; 14.9; 21.9; 28.9; 5.10; 12.10; 19.10	-Beethoven: Sonata Op. 5 N.1<br>-Mendelssohn: Albumblatt, Lied ohne Worte, Sonata D Major	-Replica of an Érard piano from 1840
