# Supplementary material for: Regulation of Emotions to Optimize Classical Music Performance: A Quasi-Experimental Study of a Cellist-Researcher
Source: Front Psychol. 2021 Apr 6;12:627601. doi: 10.3389/fpsyg.2021.627601 (PMC8056010; doi:10.3389/fpsyg.2021.627601)
Supplement: Supplementary file 2 [file Table_2.pdf]

Supplementary Material B

Average scores for emotions pre- / post-intervention per artistic event

**Emotion**	**Artistic Event**									
	**1**	**2**	**3**	**4**	**5**	**6**	**7**	**8**	**9**	**10**
**Joy**	0/6	4/7	5/5	2/3	2/5	2/7	2/9	2/7	6/8	8/10
**Enthusiasm**	3/4	5/3	5/3	3/5	3/7	3/7	3/6	3/7	4/8	9/10
**Hope**	4/5	4/4	5/6	4/5	4/7	4/7	5/9	4/8	5/8	4/10
**Relief**	0/3	0/2	0/3	0/3	0/6	0/6	2/7	1/7	5/8	9/10
**Gratitude**	1/2	1/3	1/2	1/2	1/2	3/3	3/5	2/4	4/6	3/8
**Admiration**	1/2	1/3	3/3	1/3	1/3	2/3	2/5	3/5	4/7	4/8
**Surprise**	0/1	0/2	0/1	0/0	0/1	0/1	0/0	0/0	0/0	0/0
**Pride**	0/1	2/1	1/2	1/3	0/2	0/1	0/1	1/3	2/4	3/6
**Anger**	2/2	2/1	1/0	1/1	1/1	1/1	1/0	1/0	1/0	1/0
**Contempt**	0/6	2/7	0/5	1/3	1/5	0/7	2/9	1/7	2/8	6/10
**Sadness**	3/5	0/2	0/2	0/2	0/1	2/1	0/0	1/1	1/0	7/0
**Hopelessness**	4/5	4/4	5/6	4/5	4/7	4/7	5/9	4/8	5/8	4/10
**Disappointment**	0/4	0/2	0/0	0/1	0/1	0/1	0/0	0/0	0/0	0/0
**Boredom**	1/1	2/2	1/1	0/0	0/0	0/0	0/0	0/0	0/0	0/0
**Anxiety**	6/0	5/0	4/0	3/0	1/0	2/0	1/0	0/0	1/0	0/0
**Shame/Guilt**	3/2	3/1	3/1	1/1	0/0	1/0	1/0	0/0	0/0	0/0
